# Methods to evaluate rare variants gene-age interaction for triglycerides

**DOI:** 10.1186/s12919-018-0136-7

**Published:** 2018-09-17

**Authors:** Tony Huayang Gao, Jianjun Zhang, Diaz Medina Miguelangel, Xuexia Wang

**Affiliations:** 1 Texas Academy of Mathematics & Science, University of North Texas, 1155 Union Circle #311430, Denton, TX 76203 USA; 2 Department of Mathematics, University of North Texas, 1155 Union Circle #311430, Denton, TX 76203 USA; 3 Brady Corporation, 6555 W Good Hope Rd, Milwaukee, WI 53223 USA

## Abstract

Triglycerides are an important measure of heart health. Although more than 90 genes have been found to be associated to lipids, they only explain 12 to 15% of the variance in lipid levels. Evidence suggests that age may interact with the genetic effect on lipid levels. Existing methods to detect the main effect of rare variants cannot be readily applied for testing the gene environment interaction effect of rare variants, as those methods either have unstable results or inflated Type I error rates when the main effect exists. To overcome these difficulties, we developed two statistical methods: testing of optimally weighted combination of single-nucleotide polymorphism (SNP) environment interaction (TOW-SE) and a variable weight TOW-SE (VW-TOW-SE) to test the gene environment interaction effect of rare variants by grouping SNPs into biologically meaningful SNP-sets (SNPs in a gene or pathway) to improve power and interpretability. The proposed methods can be applied to either continuous or binary environmental variables, and to either continuous or binary outcomes. Simulation studies show that Type I error rates of the proposed methods are under control. Comparing the two methods with the existing interaction sequence kernel association test (iSKAT), the VW-TOW-SE is the most powerful test and the TOW-SE is the second most powerful test when gene environment interaction effect exists for both rare and common variants. The three tests were applied to the GAW20 simulated data, among the five regions in which the main effect of common SNPs was simulated and the gene–age interaction effect was not included. As expected, none of the tests indicated positive results.

## Background

Highly heritable triglycerides (TG) [1] are an important measure of heart health. Having excess levels of TG can increase the risk of heart disease. Identified common variants only explain 12% to approximately 15% of the variance in lipid levels [2]. A substantial proportion of lipid heritability is unexplained [3]. This suggests that rare (minor allele frequency [MAF] < 1%) or intermediate variants (0.01 < MAF < 0.05) with potentially larger effect sizes or other mechanisms, such as gene–environment interactions, may play a role in explaining the substantially missing heritability.

Clear evidence shows that lipids vary by age. A handful of lipid loci with age-dependent effects were identified from candidate gene studies and genome-wide association study (GWAS) [4, 5]. However, few of these explored the role of gene–age interaction for rare and intermediate variants in lipid levels. More than 74.6% of variants are rare and intermediate variants [6], which may have a larger effect size than common variants and explain substantial proportions of lipid variance. In this GAW20 study, we attempted to detect the effect of gene–age interactions on TG for rare and intermediate variants with novel statistical methods.

Due to the allelic heterogeneity and the extreme rarity of individual variants [7], most existing methods focus on improving the power of detecting gene–environment (G × E) interactions only for individual markers, especially for common variants, and are not optimal for detecting rare variants. Although there has been interest in multiple-marker analysis by grouping single-nucleotide polymorphisms (SNPs) into biologically meaningful SNP-sets (eg, SNPs in a gene or pathway) to improve power and interpretability, the existing SNP set analysis has focused on testing for the marginal effect of a SNP set [8, 9]. Limited work has been done on testing the interactions between a SNP set and an environmental variable, especially as it pertains to rare variants. Although the SNP-set–based interaction sequence kernel association test (iSKAT) [10] can be applied to detect G × E interactions in rare variants, its power is very restricted and lacks robustness to the shape of the data in many circumstances. Motivated by the need for powerful methods to test G × E interactions for rare variants, we developed two novel methods: testing of optimally weighted combination of SNP environment interaction (TOW-SE) and a variable weight TOW-SE (VW-TOW-SE) to identify G × E interactions for SNP sets of common and/or rare variants in GWAS, exome, or next-generation sequencing data. Our simulation studies show that the Type I error rates of the proposed methods are under control. Comparing the two methods with the iSKAT, and the VW-TOW-SE is the most powerful test, TOW-SE is the second most powerful test when G × E interaction effect exists for both rare and common variants.

The Genetic Analysis Workshop (GAW) 20 “SimulationBestOneRepresentative” data includes TG levels before and after treatment with fenofibrate and genotyped genome-wide SNPs from the Genetics of Lipid Lowering Drugs and Diet Network (GOLDN) study [11]. We imputed chromosomes 1, 6, 8, 9, 10, and 17, where five major main-effect causal SNPs of TG reside. We applied the proposed methods (TOW-SE and VW-TOW-SE) and iSKAT to the unrelated individuals (sample size *n* = 246) to test TG susceptible to gene–age interactions on the five imputed genes. The main effect of common SNP was simulated in the five regions. However, the gene–age interaction effect was not included. As expected, using the proposed methods, none of the regions indicated significantly gene–age interaction effects on TG.

## Methods

Consider a sample of *n* individuals. Each individual has been genotyped at *M* variants in a genomic region (a gene or a pathway). For the *i*^*th*^ individual, denote *y*_*i*_ as the trait value (continuous or binary); *E*_*i*_ as the environmental variable (continuous or binary); *G*_*i*_ = (*g*_*i*1_, ⋯, *g*_*iM*_) as the genotypic scores at *M* variants, where *g*_*im*_ ∈ {0, 1, 2} is the number of minor alleles the *i*^*th*^ individual has at the *m*^*th*^ variant. *Z*_*i*_ denotes the potential confounder covariates.

We use the generalized linear model (GLM):


1


to model the relationship between trait values and G × E interactions *E*_*i*_*G*_*i*_, where *f* (∙) is a monotone “link” function. For a quantitative trait, *f* (∙) will be an identity link function. For a binary trait, a logit link function will be used. Coefficients of each term in Eq. (1) are denoted by *α*_0_, *a*, *β*, *Ϛ*, and *η*, respectively. To test for the G × E interaction for a SNP set of *M* SNPs is equivalent to testing the null hypothesis *H*_0_ : *β* = 0 in Eq. (1).

To test *H*_0_ : *β* = 0 in Eq. (1), we developed a score test by treating *α*_0_, *a*, *Ϛ*, and *η* as nuisance parameters. First, we adjusted both trait value *y*_*i*_ and G × E interaction *E*_*i*_*G*_*i*_ for the covariates *Z*_*i*_, the genotypic score *G*_*i*_ and the environmental variable *E*_*i*_ by applying linear regression and obtaining residuals. Denote${\overset{\sim }{y}}_i$ as the residual of *y*_*i*_ and ${\overset{\sim }{X}}_i=\left({\overset{\sim }{x}}_{i1},\cdots, {\overset{\sim }{x}}_{iM}\right)$as the residual of *E*_*i*_*G*_*i*_. Then, the relationship between${\overset{\sim }{y}}_i$ and ${\overset{\sim }{X}}_i=\left({\overset{\sim }{x}}_{i1},\cdots, {\overset{\sim }{x}}_{iM}\right)$ can be modeled by the GLM:

2 $$ f\left(E\Big({\overset{\sim }{y}}_i|{\overset{\sim }{X}}_i\Big)\right)={\beta_0}^{\ast }+{\overset{\sim }{X}}_i{\beta}^{\ast } $$

To test *H*_*o*_ : *β* = 0 in equation (1) is equivalent to test *H*_*o*_ : *β*^∗^ = 0 in equation (2). Sha et al. [12] proposed a score test to test *H*_*o*_ : *β*^∗^ = 0 in GLM. However, for rare variants’ SNP–environment interactions, the score test may lose power as a consequence of the sparse data and a large degree of freedom. To increase power by effectively using information from data, we proposed to test the G × E interactions by testing the effect of a weighted combination of SNP–environment interactions, ${\overset{\sim }{x}}_i={\sum}_{m=1}^M{w}_m{\overset{\sim }{x}}_{im}.$

To test${\overset{\sim }{x}}_i={\sum}_{m=1}^M{w}_m{\overset{\sim }{x}}_{im}$, the score test is:

3 $$ {\displaystyle \begin{array}{c}S\left({w}_1,\cdots, {w}_M\right)=n\frac{{\left({\sum}_{i=1}^n\left({\overset{\sim }{y}}_i-\overset{\sim }{y}\right)\left({\overset{\sim }{x}}_i-\overset{\sim }{x}\right)\right)}^2}{\sum \limits_{i=1}^n{\left({\overset{\sim }{y}}_i-\overline{\overset{\sim }{y}}\right)}^2{\sum}_{i=1}^n{\left({\overset{\sim }{x}}_i-\overline{\overset{\sim }{x}}\right)}^2}\\ {}=n\frac{{\left({\sum}_{m=1}^M{w}_m{\sum}_{i=1}^n\left({\overset{\sim }{y}}_i-\overline{\overset{\sim }{y}}\right)\left({\overset{\sim }{x}}_{im}-{\overline{\overset{\sim }{x}}}_m\right)\right)}^2}{\sum \limits_{i=1}^n{\left({\overset{\sim }{y}}_i-\overline{\overset{\sim }{y}}\right)}^2{\sum}_{i=1}^n{\left({\overset{\sim }{x}}_i-\overline{\overset{\sim }{x}}\right)}^2}\end{array}} $$

It reaches its maximum ${S}_o\left({w}_1^0,\cdots, {w}_M^0\right)=n\sum \limits_{i=1}^n\left({\overset{\sim }{y}}_i-\overline{\overset{\sim }{y}}\right)\left({\overset{\sim }{x}}_i^0-{\overline{\overset{\sim }{x}}}^0\right)/{\sum}_{i=1}^n{\left({\overset{\sim }{y}}_i-\overline{\overset{\sim }{y}}\right)}^2$ when ${w}_m^0=\frac{\sum \limits_{i=1}^n\left({\overset{\sim }{y}}_i-\overline{\overset{\sim }{y}}\right)\left({\overset{\sim }{x}}_{im}-{\overline{\overset{\sim }{x}}}_m\right)}{\sum \limits_{i=1}^n{\left({\overset{\sim }{x}}_{im}-{\overline{\overset{\sim }{x}}}_m\right)}^2}$; ${\overset{\sim }{x}}_i^0={\sum}_{m=1}^M{w}_m^0{\overset{\sim }{x}}_{im},$ as rare variants are essentially independent. Thus, ${w}_m^0$ is the optimal weight. We define ${T}_{T- SE}=\sum \limits_{i=1}^n\left({\overset{\sim }{y}}_i-\overline{\overset{\sim }{y}}\right)\left({\overset{\sim }{x}}_i^0-{\overline{\overset{\sim }{x}}}^0\right)$ as the statistic to Test the effect of the Optimally Weighted combination of SNP-Environment interactions (TOW-SE), which is equivalent to ${S}_o\left({w}_1^0,\cdots, {w}_M^0\right)$ when we use a permutation test to evaluate *p*-values.

We analytically derive optimal weights for TOW-SE. The optimal weight ${w}_m^0$ will put a big weight to SNP–environment interactions that have strong association with the trait of interest and also adjust the direction of the association. Moreover, it will put big weights to SNP–environment interactions with small variations that are often rare variants. iSKAT assigns weights to variants based on the MAFs via a beta function. It put decent nonzero weights for variants with MAF in (0.01, 0.05). If MAFs of causal variants are not in the range of (0.01, 0.05), iSKAT will be less powerful than TOW-SE. TOW-SE targets rare variants and may lose power when testing G × E effects of both rare and common variants.

To test for the G × E interaction of both rare and common variants, we propose variable weight TOW-SE (VW-TOW-SE). We divide variants into rare and common. Let *T*_*r*_ and *T*_*c*_ denote the test statistic of TOW-SE for rare and common variants, respectively. Let ${T}_{\lambda }=\lambda \frac{T_r}{\sqrt{\mathit{\operatorname{var}}\left({T}_r\right)}}+\left(1-\lambda \right)\frac{T_c}{\sqrt{\mathit{\operatorname{var}}\left({T}_c\right)}}.$ Denote *p*_*λ*_ as the *p-*value of *T*_*λ*_. The test statistic of VW-TOW-SE is defined as *T*_*VW* − *T* − *SE*_ = *min*_0 ≤ *λ* ≤ 1_*p*_*λ*_. We will use permutations to evaluate *p-*values of both *T*_*T* − *SE*_ and *T*_*VW* − *T* − *SE*_.

### Simulations

Following the simulation setting in Lin et al. [10], we conducted simulation studies using the GAW17 empirical mini-exome sequenced data. The data set contains genotypes of 697 unrelated individuals on 3205 genes. Research shows that SNP rs11583200 on gene *ELAVL4* is associated with body mass index [13] and rare variants on gene *ELAVL4* are associated with the quantitative trait Q1 in the GAW17 data [14]. Therefore, we chose gene *ELAVL4* in our simulation study. There are 10 variants on gene *ELAVL4* of which 8 are rare variants and 2 are common variants. The rare variants threshold was chosen as 0.01. We use the program *fastPHASE* [15] to infer haplotypic phase for the 697 individuals and calculate haplotype frequencies. To generate the genotype of an individual, we generate 2 haplotypes according to the haplotype frequencies. The quantitative trait was generated using the following model:

4 $$ Y=0.5{Z}_1+0.5{Z}_2+E{\alpha}_1+{\boldsymbol{G}}^{\boldsymbol{T}}{\alpha}_2+\boldsymbol{E}{\boldsymbol{G}}^{\boldsymbol{T}}\beta +\boldsymbol{E}{\boldsymbol{G}}_{\boldsymbol{c}}{B}^c+\epsilon $$

where *Z*_1_~*N*(0, 1); *Z*_2_~Binomial(1, 0.5) and *ϵ*~*N*(0, 1).The environmental variable *E* is assumed to be continuous following standard normal distribution and we set *α*_1_ = 0.015; ***EG*** is the rare variants G × E interaction and ***EG***_***c***_ is one common variant G × E interaction.

We consider two scenarios: (a) with main effect and (b) without main effect in the model (4). When there are no main effects, we set the magnitudes of vector *α*_2_ = 0.3 for each element and their signs are randomly sampling from (−1, 1). When there are no main effects, we set *α*_2_ = 0. To evaluate the Type I error, we set *β* and *β*^*c*^ all to 0. To evaluate power, we vary the number of non-zero elements *β*_*j*_ in *β*. We set the magnitude of the nonzero *β*_*j*_ as |*β*_*j*_| = *c*, and increase *c* from 0.02 to 0.1. Of the *β*_*j*_, 50% are positive. *β*^*c*^ is positive and twice the magnitude of *β*_*j*_. The sample size is 2000 for each scenario. *P* values are estimated by 10,000 permutations. The Type I error rates and power are evaluated using 1000 replicated samples.

### GAW20 data analysis

We applied TOW-SE, VW-TOW-SE, and iSKAT to the GAW20 “SimulationBestOneRepresentative” data, which includes TG levels before and after treatment with fenofibrate and genotyped genome-wide SNPs from the GOLDN project [11]. We imputed chromosomes 1, 6, 8, 9, 10, and 17 with *minimac2* software [16]. Five major main effect causal SNPs (rs9661059, rs736004 [*LYRM4*], rs1012116, rs10828412, and rs4399565 [*HS3ST3A1*]) of TG reside on chromosomes 1, 6, 8, 9, 10, and 17, respectively. The 1000 Genomes project haplotypes integrated phase I served as the reference panel. It includes 1092 individuals. Our analysis was based on 246 unrelated individuals. Both pre-treatment and post-treatment TG values were provided for two visits. We used the log ratio of the pre-treatment mean and the post-treatment mean as the phenotype trait in our G × E interaction analysis. For individuals who did not have two visits, we just used the existing value. The median age is 64 years (range: 28–83 years). We used the centered age in our analysis. The median of the TG ratio is 1.54 (range: 0.72–4.24). We excluded 8 individuals from our analysis because of completely missing post-treatment TG values.

We evaluated the performance of TOW-SE, VW-TOW-SE, and iSKAT by testing G × E interaction effect on TG for the aforementioned 5 regions. Each region consists of 10 SNPs, and the fifth SNP in each region is the major main effect causal SNP of TG. Additionally, we assessed the effect of region size on the power of the tests using the region of rs4399565.

## Results

**Fig. 1:**
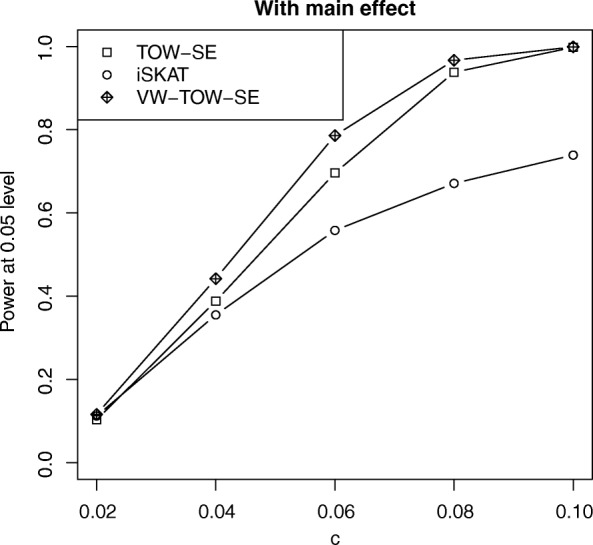
Power comparisons of three tests (TOW-SE, iSKAT, VW-TOW-SE) for *n* = 2000 at α = 0.05 level of significance for testing both rare and common variant G × E interaction effects on a continuous outcome with main effect

**Fig. 2:**
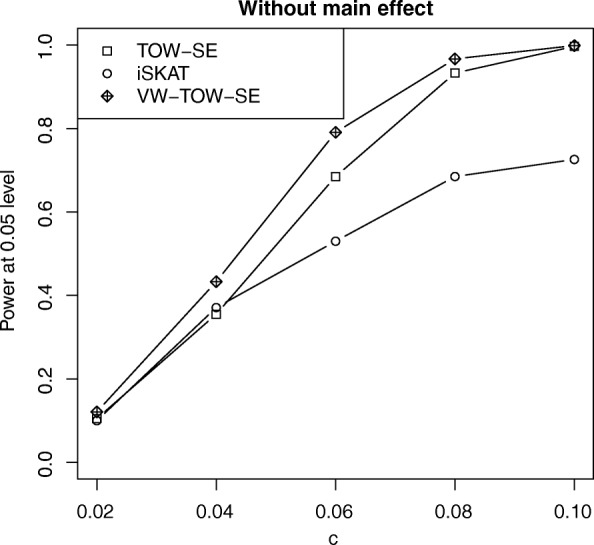
Power comparisons of three tests (TOW-SE, iSKAT, VW-TOW-SE) for *n* = 2000 at α = 0.05 level of significance for testing both rare and common variant G × E interaction effects on a continuous outcome without main effect

Table 1 shows that the Type I error rates of all the three methods are under control. Power comparisons of the three tests (VW-TOW-SE, TOW-SE, and iSKAT) for different values of G × E effect for rare and common variants are given in Fig. 1 (with main effect) and Fig. 2 (without main effect). The power of the three tests increases as the effect size increases. When there is a G × E interaction effect for both rare and common variants, VW-TOW-SE is the most powerful test and TOW-SE is the second most powerful test. Table 2 shows that the median of the MAF ranges from 0.003 to 0.013 in the 5 regions. When we apply the three tests to test G × E interaction effect, under Bonferroni correction, none of the regions are significantly associated with TG. Table 3 suggests that all of the three methods perform better when the region size is larger.

**Table 1:** Type I error rates for both rare and common variants in the presence of main effects (*top panel*) and in the absence of main effects (*bottom panel*) for *n* = 2000

	α-level	TOW-SE	iSKAT	VW-TOW-SE
With main effect				
*n* = 2000	0.050	0.050	0.055	0.059
	0.010	0.011	0.012	0.015
	0.001	0.000	0.001	0.000
Without main effect				
*n* = 2000	0.05	0.051	0.061	0.056
	0.01	0.011	0.013	0.009
	0.001	0.000	0.003	0.001

**Table 2:** Results of testing G × E interaction in the five causal regions using the three methods

Region name (SNP set)	Median of MAF (range)	*P*_*TOW-SE*_	*P*_*iSKAT*_	*P*_*VW-TOW-SE*_
rs736004	0.013 (0.001–0.387)	0.020	0.022	0.019
rs1012116	0.015 (0.002–0.167)	0.024	0.030	0.021
rs4399565	0.006 (0.001–0.449)	0.022	0.023	0.019
rs9551059	0.007 (0.001–0.275)	0.031	0.030	0.037
rs10828412	0.003 (0.001–0.394)	0.100	0.099	0.095

**Table 3:** Region size effect analysis for the three methods based on the region of rs4399565

Region size	Median of MAF (range)	*P*_*TOW-SE*_	*P*_*iSKAT*_	*P*_*VW-TOW-SE*_
10 SNPs	0.006 (0.001–0.449)	0.022	0.023	0.019
20 SNPs	0.008 (0.001–0.449)	0.014	0.018	0.015
30 SNPs	0.008 (0.001–0.449)	0.013	0.014	0.012

## Discussion

The computation time for TOW-SE and VW-TOW-SE using 10,000 permutations for analyzing 1000 individuals in a region that includes 50 SNPs is 9 s and 20 s, respectively. Suppose a whole genome sequencing data with 13,498,188 SNPs, TOW-SE will take 674 h (28 days) to conduct a whole genome analysis. The effect analysis of the region size suggests that the three methods will perform better when the region size is larger. However, the larger the region size, the higher chance for collinearity to appear in the region, which makes the computation more complex. To minimize the problem of collinearity, we recommend a region size between 10 SNPs and 30 SNPs when we apply the proposed methods to a genome-wide scan.

## Conclusions

In summary, we developed two novel statistical methods: TOW-SE and VW-TOW-SE by grouping SNPs into biologically meaningful SNP-sets, which improved power and interpretability. Simulation studies show that the proposed methods yielded well-controlled Type I error rates under all study conditions. When gene environment interaction effect exists for both rare and common variants, VW-TOW-SE is the most powerful test, TOW-SE is the second most powerful test, and iSKAT is the least powerful test.
